# Reply to Cho, P.S.P. Comment on “Kardos et al. Efficacy and Safety of a Single Ivy Extract Versus Two Herbal Extract Combinations in Patients with Acute Bronchitis: A Multi-Center, Randomized, Open-Label Clinical Trial. *Pharmaceuticals* 2025, *18*, 754”

**DOI:** 10.3390/ph19040593

**Published:** 2026-04-08

**Authors:** Peter Kardos, Justus de Zeeuw, Inga Trompetter, Yuliya Ilieva, Simon Braun

**Affiliations:** 1 Lung Centre Maingau, 60316 Frankfurt am Main, Germany; kardos@lungenzentrum-maingau.de; 2 Pneumologische Praxis, 51105 Köln, Germany; 3 Engelhard Arzneimittel GmbH & Co. KG, 61138 Niederdorfelden, Germany; i.trompetter@engelhard.de (I.T.); y.ilieva@engelhard.de (Y.I.)

Firstly, we would like to thank the author of the letter [1] for their critical examination of our publication, giving us the opportunity to further consider our study design and the comparator drugs.

Prior to its publication, the manuscript underwent a rigorous peer-review process, the details of which have also been published (see https://www.mdpi.com/1424-8247/18/5/754/review_report, (accessed on 11 March 2026). Among other things, one of the reviewers involved in the process confirmed that there were no objections to the design of the study or its discussion. We are pleased to address the concerns that have been raised point by point.

*Concern*: Open-label design

*Answer*: Indeed, in the absence of blinding, both patient perception and investigator evaluation may have been influenced by knowledge of the treatment received. However, all the investigated products have demonstrated their efficacy in randomized, placebo-controlled trials [2,3,4,5] and are well-known cough remedies among patients in Germany, as well as among the investigators. Against this background, it may be that among the study participants and participating investigators, there are those who, for whatever individual reasons, prefer one cough medicine over the others. However, there is no evidence that such preferences had an exclusive or predominant effect in favor of any treatment arm. Rather, distortions to the detriment of Ivy mono are plausible, as combination preparations are often assumed to be more effective. At the very least, potential preferences were distributed evenly among the three equally well-known cough medicines included in the study and sufficiently large comparison groups minimized the likelihood of selective influence on the results. Additionally, the demonstrated statistical significance against the Ivy/Thyme comparator—but not against the Thyme/Primrose comparator—indicates that no Ivy mono bias affected the study results.

Due to the different dosages, a triple-dummy design in our case would have required five times daily intake of three medications prior to meals, with liquid intake with sugar or hot tea, which seems unduly burdensome in the context of acute bronchitis and would have introduced an adherence bias. Please note that the examples provided in the letter to substantiate the simplicity of triple-dummy designs were, in fact, studies conducted with patients suffering from asthma or Chronic Obstructive Pulmonary Disease (COPD), with one or two daily intakes of each preparation [6,7], or that used double-dummy designs [8,9], but not complex triple-dummy designs.

*Concern*: Unequal randomization

*Answer*: Unequal randomization ratios were deliberately selected and prospectively defined in the study protocol as such setups are common in statistical experimental design. To eliminate potential statistical bias and ensure the targeted statistical power of 90%, a large total number of at least 300 patients was specifically predefined. The number of patients was therefore sufficient within the two comparison groups, with each group comprising over 90 patients, to enable the derivation of robust statistical conclusions within the framework of this confirmatory study design. In general, unbalanced sample sizes do not undermine the validity of the significance statements (*p*-values) generated by the applied statistical tests.

*Concern*: Endpoints

*Answer*: We agree that both the Bronchitis Severity Score (BSS) and Visual Analog Scale (VAS) are common and appropriate parameters in studies investigating bronchitis. Indeed, there is no anchor- or distribution-based Minimal Clinically Important Difference (MCID) available for the BSS. Therefore, we applied 15% change in any scale, as suggested by the German Institute for Quality and Efficiency in Healthcare (IQWiG, the German independent scientific body that evaluates medical evidence to inform healthcare decisions, sharing similar goals as the National Institute for Health and Care Excellence (NICE) in UK). In a methodology paper, the IQWiG reviewed systematically researched reviews on MCIDs and identified 15% of the range of the respective scales as a plausible threshold value for a sufficiently reliable, noticeable change that can be applied if no validated MCID is available for the scale [10]. Please note that this threshold is rigid and may even overestimate the MCID, especially when compared to other cough-related questionnaires like the St. George’s Respiratory Questionnaire, Leicester Cough Questionnaire, and COPD Assessment Test, where much smaller changes in the range of 4–7% have been determined as respective MCIDs [11,12,13].

We thank Dr. Cho for the valuable suggestion to consider including additional assessments such as quality-of-life assessments in future studies. At the same time, we would like to point out that global efficacy and global tolerability have been assessed and thus parameters beyond cough-related endpoints are already included in our panel of assessed parameters (see Figure 6 and Supplementary Figures S1 and S2, Kardos, P.; de Zeeuw, J.; Trompetter, I.; Braun, S.; Ilieva, Y. Efficacy and Safety of a Single Ivy Extract Versus Two Herbal Extract 97 Combinations in Patients with Acute Bronchitis: A Multi-Center, Randomized, Open-Label Clinical Trial. *Pharmaceuticals (Basel)* 98 **2025**, 18, 754. doi:10.3390/ph18050754.). Also, these explorative results align well with and thus further confirm the results from the primary and secondary endpoints.

*Concern*: Incomplete reporting

*Answer*: Our study recorded numerous parameters, including global efficacy assessment, global tolerability assessment, VAS, verbal category descriptive score, and BSS. Since the manuscript already contains six figures, two supplementary figures, and two tables, it would be impractical to present all results as figures and tables, or even to present different aspects of certain measurement parameters in multiple figures, so we respectfully decline this suggestion. This would have dramatically increased the length of the manuscript at the expense of readability.

We agree that Figure 5 (Kardos, P.; de Zeeuw, J.; Trompetter, I.; Braun, S.; Ilieva, Y. Efficacy and Safety of a Single Ivy Extract Versus Two Herbal Extract 97 Combinations in Patients with Acute Bronchitis: A Multi-Center, Randomized, Open-Label Clinical Trial. *Pharmaceuticals (Basel)* 98 **2025**, 18, 754. doi:10.3390/ph18050754.) underscores the global trend in favor of Ivy, but at the same time does not guarantee millimeter-precise readability of the VAS. We would like to express our gratitude for pointing out that an MCID of 17 mm was determined for the VAS in acute cough [14] and confirm that −18.86 mm was achieved for Ivy already on day 2, while this was not yet the case for Ivy/Thyme with −9.65 mm and Ivy/Primrose with −13.06 mm at day 2. Therefore, these results are in line with our findings presented in Figure 4a (Kardos, P.; de Zeeuw, J.; Trompetter, I.; Braun, S.; Ilieva, Y. Efficacy and Safety of a Single Ivy Extract Versus Two Herbal Extract 97 Combinations in Patients with Acute Bronchitis: A Multi-Center, Randomized, Open-Label Clinical Trial. *Pharmaceuticals (Basel)* 98 **2025**, 18, 754. doi:10.3390/ph18050754.) for the BSS, showing that the threshold of MCID (3.15 points on the BSS) was surpassed on day 2 for the Ivy group but not yet for the Ivy/Thyme or Ivy/Primrose groups. An effect corresponding to a one-day reduction in symptom duration, supported by statistical significance and clinical relevance, may be considered meaningful in routine clinical practice—particularly in the context of self-limiting, acute bronchitis, which typically resolves within a few days.

*Concern*: Reference to independent placebo-controlled trials to underscore validity

*Answer*: Our data are well in-line with the data from previous double-blind, placebo-controlled trials. We believe that omitting this important information from the discussion would have been a significant shortcoming, especially since contextualizing the results of current work with previous research is a common practice that we consider absolutely essential. Particularly, we showed that results from our head-to-head trial are largely consistent with both onset of treatment effects and even absolute BSS values. We moreover do not consider the respective placebo-controlled trials to be substantially different. While minor methodological differences exist—such as varying BSS entry thresholds (5 points in [4,5] vs. 10 points in [2,3,15]) and visit schedules with variable resolution during the treatment period—the key design features are highly comparable. Across studies, the patient population is similar (adults with acute bronchitis), change in BSS is a primary endpoint [3,5,15] or secondary endpoint [2,4], baseline BSSs are comparable (moderate to severe acute bronchitis according to [16]), and assessment timepoints are broadly aligned, including baseline (day 0), interim assessments (e.g., day 4), and end-of-treatment visits (day 7–10).

Taken together, the randomized, comparator-controlled design and the use of subjective endpoints are important considerations when interpreting the results, with the open-label nature of the trial representing the primary design-related limitation. As with any clinical trial, generalizability beyond the studied population may be limited. Nonetheless, the trial provides a valuable contribution to understanding the uniqueness of single-plant extracts and their clinical efficacy.
